# Development of an emergency airway response system for COVID-19 at a tertiary care hospital in resource limited country

**DOI:** 10.12669/pjms.39.1.5689

**Published:** 2023

**Authors:** Faisal Shamim, Muhammad Faisal Khan, Khalid Samad, Asad Latif

**Affiliations:** 1 Faisal Shamim, FCPS, Associate Professor, Department of Anaesthesiology, Aga Khan University Hospital, Karachi, Pakistan; 2 Muhammad Faisal Khan, FCPS, Assistant Professor, Department of Anaesthesiology, Aga Khan University Hospital, Karachi, Pakistan; 3 Khalid Samad, FCPS, Associate Professor, Department of Anaesthesiology, Aga Khan University Hospital, Karachi, Pakistan; 4 Asad Latif, MD, MPH, Associate Professor and Chairperson, Department of Anaesthesiology, Aga Khan University Hospital, Karachi, Pakistan

**Keywords:** COVID-19, Tracheal Intubation, Aerosol Generating Procedure, Guidelines, emergency airway response system

## Abstract

The ongoing coronavirus (COVID-19) infection causes severe respiratory dysfunction and has become an emergent issue for worldwide healthcare due to highly transmissible and contagious nature. Aerosol generating procedures such as tracheal intubation is of particularly high risk. This mandates some advice on processes and techniques required to protect staff and uniform approach during airway management. We hereby share our experience in development of an emergency response system to deal with COVID airway management at a frontline hospital which particularly consider the local demands and resources. This includes a change in working dynamics with 24/7 consultant coverage for emergent or urgent tracheal intubation of COVID patients at non-operating room locations. Other steps include prepackaging intubation baskets, availability of videolaryngoscope, standard personal protective equipment including powered air purifying respirator, and use of modified intubation checklist.

The novel coronavirus, COVID-19, has caused a pandemic of previously unimaginable proportions. Highly infective, virus has given significant burden of morbidity and mortality, primarily due to hypoxic respiratory failure.^1^ Those unfortunate enough to suffer from the severe form of the disease need airway interventions such as tracheal intubation and invasive mechanical ventilation to have any chance of survival. This leads to certain physician groups like anaesthesiologist, intensivists, and emergency physicians being at particularly high risk of getting infected due to the aerosolization of the virus during intubation and the provision of ongoing respiratory support.^2^ It is suggested that individual departments should establish and mandate a standardized approach to airway management. At the start of pandemic, little literature existed to guide management and bulk of guidance is based on expert recommendations, case reports and case series. The Aga Khan University Hospital is one of the frontline responders during this calamity in managing such patients. We focus this special communication on the development and implementation of an emergency airway response system for COVID-19 patients.

## General and structural considerations:

**Fig.1:**
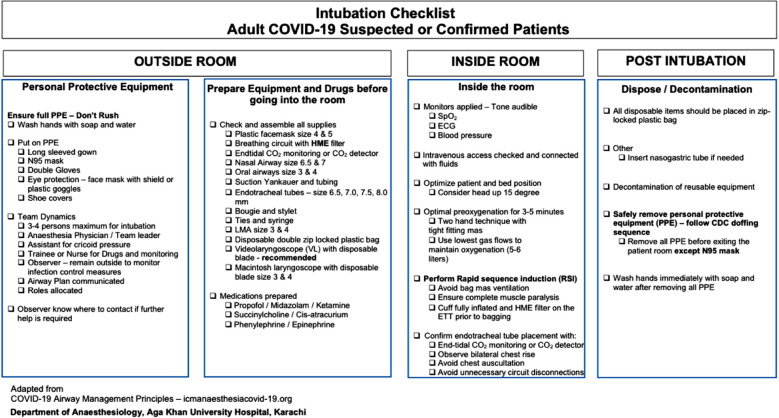
A checklist for use during intubations of COVID-19 patients as a practical bedside tool for clinicians.

In the first step, we reviewed few guidelines published by international societies.^3^ We looked at our resources, available equipment with its sustainable supplies and team dynamics. Next, we made clinical practice guidelines with the scope and purpose to assist clinicians and staff involved in airway management of COVID-19 patients (supplementary material). This document does not discuss when to intubate patients, treatment and intensive care ventilatory strategies. A checklist was also made containing all elements of guidelines and consider a minimum mandatory requirement when a suspected or confirmed COVID patient requires intubation (Fig.1). Our aim of using such checklist in clinical practice is to ensure that patient, equipment, and team-related factors are all addressed prior to the procedure. This has been shown that intubation checklists and care bundles when used in critical situations improve patient safety, reduce mortality and complication rates.^4^

Furthermore, we made personal protective equipment (PPE) prepared packs and COVID intubation baskets whose information is circulated through email. The purpose is to provide universal and most appropriate PPE for airway managers. The components of PPE packs are a cap, head cover, a facemask with eye shield, full sleeve waterproof surgical gown, two pairs of gloves (combination of sterile and non-sterile) and shoe covers. Powered Air Purifying Respirator (PAPR) is also a part of intubation basket. To understand proper donning and doffing procedure, the centers for disease control (CDC) document was printed and kept one copy in each PPE pack.

## Formulation, communication and consultation with airway team:

As suggested by all societies and guidelines that COVID intubation should be done by most experienced practitioner, we made changes to our working dynamics and roster as soon as we received our first patient in mid of March, 2020. Since then, only consultant anaesthetist have been doing emergent or urgent COVID intubations outside operating theaters and providing coverage 24/7. ICU consultant is primarily responsible in daytime and two consultants stayed in house during on calls. A dedicated pager number from hospital communication has been assigned for COVID intubation rush calls and three pagers were allocated to ICU, on call consultants and anaesthetic technician who is responsible to bring intubation baskets, PAPR and PPE packs to desired location.

## Principles of response team during intubation:

We are describing airway management of our first patient who was a 50 year old male with polymerase chain reaction (PCR) tested positive COVID-19 admitted to intensive care unit. The purpose of this description is to provide information about universal principles and how it works during every airway response system whenever a COVID emergency intubation call was generated. Our patient had bilateral pneumonia, ARDS and severe hypoxemia despite optimal management. We decided to intubate him on urgent basis and started preparation by following our departmental guidelines. This included assembling all necessary supplies and equipment outside the room, formulate and communicate airway plan to team members, assign roles, drawn medications into syringes and took complete personal protective equipment. A minimum staff of four members including two consultants and two assistants were present in a negative pressure room. An observer was kept outside to monitor the whole process for further improvement. Preoxygenation was carried out by tight fitting mask and two hand technique with 100 % oxygen for five minutes. Rapid sequence induction with cricoid pressure accomplished using injection Midazolam 2mg, Propofol 150mg and Succinylcholine 100 mg. Laryngoscopy with i-view disposable videolaryngoscope (Intersurgical Ltd, Crane House, Molly Millars Lane, Wokingham, Berkshire, RG41 2RZ, UK) and size 8.0 mm endotracheal tube passed. The ETT cuff inflated first followed by ETCO_2_ detector placement between ETT and HME filter and then patient was ventilated through re-breathing bag with low tidal volume. All these steps were taken to reduce viral aerosolization. ETT placement is confirmed by change in color on ETCO_2_ detector and bilateral chest rise, avoided chest auscultation by stethoscope. After initiation of mechanical ventilation, we undertook the doffing procedure by consulting the CDC printed document available at bedside. All disposable equipment was placed in a zip locked bag for disposal and PPE removed in patient’s room except N95 mask.

## Improvements in the system and going forward:

Since our first case, we are in continuous process to evaluate and have suggested few more things. Initially we proposed two consultants to handle airway management but then it is realized that one consultant should go into the room and the second will be stand by to minimize exposure. The face mask with eye shield sometimes becomes foggy impairing the vision, so now goggles and visors are available. Initially, we started with N95 but quite early, Powered Air Purifying Respirator (PAPR), a battery-operated device equipped with a hood or helmet, breathing tube, high efficiency particulate filter (HEPA) and powered blower, have been arranged specially for anaesthesiologist on COVID intubation call. A bulk of reusable respirators with filters were also made available for other healthcare workers. Other hospitals in the country have also adopted majority of these steps to reduce virus exposure during airway management. There have been no reports of designated PAPR use from under-resourced health systems. Because rechargeable batteries are so expensive, studies have shown that PAPR storage and use are the most economically disadvantageous options.^5^ In developing countries, intubating physician mostly used reusable respirator or N95 mask along with goggles. Locations outside operating room pose another challenge as staff are not familiar with specific precautions to take during intubation like handing\taking over the equipment and dispose them in a safely manner. This needs education and training, and we are proposing simulation-based workshops with real scenarios to better understand the dynamics during aerosol generating procedures like intubation. Haq et al. did a survey about potential barriers amongst healthcare professionals of Pakistan for managing COVID patients and emphasized the importance of formal training regarding infection control measures.^6^ There are widespread videos/pictures on anaesthesia forums about additional barriers including placing the patient under a plastic sheet or under a rigid plastic box with arm holes. This is also under consideration but we do not recommend such methods as they require careful selection of risks of adding complexity. In few cases, the authors have used a clear plastic sheet which is less intrusive for airway management and may be effective in reducing droplet dispersal. Almost every society and association have recommended videolaryngoscope (VL) with disposable blade to reduce staff exposure to airway secretions.^3^ While using disposable VL in initial 3-4 cases which has enormous cost, now we have moved to McGrath videolaryngoscope with disposable blades (Medtronic parkway Minneapolis, USA). A retrospective information is collected from anaesthesiologist who did intubations in COVID patients about the ease of intubation, glottic view and use of accessory maneuvers/adjuncts (external laryngeal manipulation, stylet, bougie) with McGrath videolaryngoscope. None have reported significant problems except the screen resolution which is more whitish and brighter.

**Fig.2:**
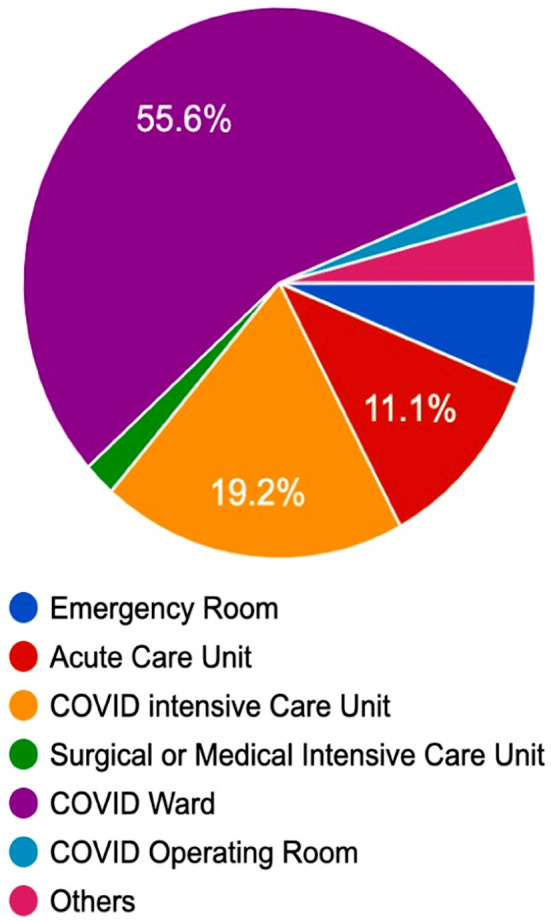
Distribution of number of tracheal intubations (%) in COVID-19 patients at various hospital locations.

To date, anaesthesia airway response system in our hospital has responded and performed over two hundred airway management/tracheal intubation episodes at non-operating room locations (acute care unit [ACU], intensive care unit [ICU], COVID ward and sometimes emergency department) by following the same principles (Fig.2). The airway management is aimed at minimizing disconnection, aerosolization and exposure of staff to viral transmission.^7^ The decision to make pre-packed PPE kits and intubation baskets along with designated people carrying pagers in day and night have shown a consistent management and positive outcome.

## CONCLUSION

As demonstrated through this special communication, corona virus outbreak is a sweeping and unprecedented challenge all over world. There is a need of organized response systems in emergency airway management of these patients at every tertiary care public and private sector hospital. The key factors include that COVID patient tracheal intubation by most experienced anaesthesia personnel, PPE of an airborne level, limit number of staff as much as possible during the procedure, clear and closed loop communication, and a robust infrastructure to meet supply and demand.

### Disclaimer:

This is to certify that the manuscript has been read and approved by all the authors, the requirements for authorship have been met, and each author believes that the manuscript represents honest work.

### Authors’ Contributions:

**FS** and **MFK** conceived and designed

**FS** and **KS** did data collection and manuscript writing

**AL** did statistical analysis and manuscript editing

**FS** takes the responsibility and is accountable for all aspects of the work in ensuring that questions related to the accuracy or integrity of any part of the work are appropriately investigated and resolved.
